# Large Electrocaloric Effect in Lead-Free (Ba_0.85_Ca_0.15_)(Zr_0.1_Ti_0.9_)O_3_ Ceramics Prepared via Citrate Route

**DOI:** 10.3390/ma10091093

**Published:** 2017-09-18

**Authors:** Jing Shi, Rongfeng Zhu, Xing Liu, Bijun Fang, Ningyi Yuan, Jianning Ding, Haosu Luo

**Affiliations:** 1 School of Materials Science and Engineering, Jiangsu Collaborative Innovation Center of Photovolatic Science and Engineering, Jiangsu Province Cultivation Base for State Key Laboratory of Photovoltaic Science and Technology, National Experimental Demonstration Center for Materials Science and Engineering, Changzhou University, Changzhou 213164, China; shijing@ccit.js.cn (J.S.); izhurongfeng@outlook.com (R.Z.); nyyuan@cczu.edu.cn (N.Y.); 2 Institute of Electronic and Electrical, Changzhou College of Information Technology, Changzhou 213164, China; 3 School of Materials Science and Engineering, Tongji University, Shanghai 201804, China; lxferro@163.com; 4 School of Material Science and Engineering, Jiangsu University, Zhenjiang 212013, China; 5 Key Laboratory of Inorganic Function Material and Device, Chinese Academy of Sciences, Shanghai 201800, China

**Keywords:** lead-free BCZT piezoceramics, citrate method, electrocaloric effect, Raman spectroscopy

## Abstract

The 1 wt % Li-doped (Ba_0.85_Ca_0.15_)(Zr_0.1_Ti_0.9_)O_3_ (BCZT-Li) ceramics prepared by the citrate method exhibit improved phase purity, densification and electrical properties, which provide prospective possibility to develop high-performance electrocaloric materials. The electrocaloric effect was evaluated by phenomenological method, and the BCZT-Li ceramics present large electrocaloric temperature change ∆*T*, especially large electrocaloric responsibility *ξ* = ∆*T*_max_/∆*E*_max_, which can be comparable to the largest values reported in the lead-free piezoelectric ceramics. The excellent electrocaloric effect is considered as correlating with the coexistence of polymorphic ferroelectric phases, which are detected by the Raman spectroscopy. The large *ξ* value accompanied by decreased Curie temperature (around 73 °C) of the BCZT-Li ceramics prepared by the citrate method presents potential applications as the next-generation solid-state cooling devices.

## 1. Introduction

Facing increasingly serious energy crisis and environmental protection requirements, electrocaloric materials, using adiabatic entropy and temperature change in polar materials under external electric field and presenting promising versatile applications in energy-efficient microelectronic and solid-state cooling devices, have attracted intensive research attention [1,2]. Large electrocaloric effect can be obtained in the ferroelectric materials with perovskite structure since their polarization exhibits great dependency on temperature approaching to the ferroelectric phase transition temperature (*T_C_*) especially with the compositions around the morphotropic phase boundary (MPB) [3,4]. It is well known that the MPB compositions are formed typically by relaxor ferroelectrics combining with normal ferroelectrics in special composition region, which present giant dielectric and piezoelectric responses due to engineered domain configuration, polarization rotation mechanism, adaptive intermediate monoclinic/orthorhombic ferroelectric phase and/or polar nanoregion [5,6,7,8]. Therefore, ferroelectric materials with the MPB compositions provide prospective possibility to develop high-performance electrocaloric materials.

Recently Mischenko et al. discovered a magnitude higher electrocaloric effect in the PbZr_0.95_Ti_0.05_O_3_ thin films around the *T_C_* temperature [1], which ignited active research interest on various ferroelectric materials for commercial exploiting [9,10,11,12]. Although readily large electrocaloric effect was fulfilled in the Pb-containing ferroelectrics [13,14,15,16], their electrocaloric strength |∆*T*|/|∆*E*| reduced significantly due to the applied ultrahigh external electric field. Furthermore, such materials were restricted by the European Union regulation and by many countries due to the toxicity of lead [14,15,16], then, searching for environmental friendly lead-free electrocaloric ferroelectrics became extremely urgent.

Another existing dilemma ferroelectrics present is maximum electrocaloric response around the *T_C_* temperatures influenced by the conjugate applied electric field [11], which limits the lead-based ferroelectrics potential electrocaloric cooling applications since their *T_C_* is relatively high [14,15,16]. To meet the requirements of novel generation solid-state cooling devices, electrocaloric materials presenting large electrocaloric effect around room-temperatures are highly desirable for various applications [1,2,3,4,11].

The discovery of the ultrahigh piezoelectric performance lead-free Ba(Zr_0.2_Ti_0.8_)O_3_-*x*(Ba_0.7_Ca_0__.3_)TiO_3_ (BZT-BCT) ceramics with relatively low *T_C_* (*d*_33_ = 620 pC/N and *T_C_* = 93 °C) provides potential candidates in practical electrocaloric applications [17]. However, significant investigations have been carried out on ceramics processing by the solid-state sintering method in order to increase *T_C_* and understand the mechanisms of ferroelectric phase transition and piezoelectric response origins rather than electrocaloric effects [18,19,20,21]. It is well-known that ceramics synthesis methods and processing conditions, and chemical doping influence phase structure formation and microstructure morphology [22,23], which exert great effects on the electrical properties of the synthesized ceramics via the intrinsic factors correlated with phase structure and lattice distortion and extrinsic factors correlated with microstructure morphology and domain configuration [24,25]. Therefore, 1 wt % Li-doped (Ba_0.85_Ca_0.15_)(Zr_0.1_Ti_0.9_)O_3_ (BCZT-Li) ceramics were prepared by the citrate method and the effects of Li doping and citrate method were reported [26,27]. The BCZT-Li ceramics with the MPB composition prepared by the citrate method exhibit enhanced ferroelectric property and relatively large ∆*T* with decreased *T_C_* temperature (around 73 °C) approaching to the room-temperature, presenting potential applications as the next-generation solid-state cooling devices. In this study, the electrocaloric performance of the BCZT-Li ceramics was investigated by the phenomenological model using the thermodynamic relation and the ferroelectric phase transition enhanced electrocaloric effect was discussed.

## 2. Materials and Methods 

1 wt % Li-doped (Ba_0.85_Ca_0.15_)(Zr_0.1_Ti_0.9_)O_3_ (BCZT-Li) polycrystalline ceramics were prepared by the citrate method using LiNO_3_, Ba(NO_3_)_2_, Ca(NO_3_)_2_·4H_2_O, Zr(NO_3_)_4_·5H_2_O and C_16_H_36_O_4_·Ti as raw materials, and the detailed experimental procedures were described elsewhere [26,27]. Mass specific heat *C_p_* was measured by a PerkinElmer Pyris DSC 8500 (PerkinElmer Inc., Akron, OH, USA) differential scanning calorimeter (DSC). Temperature dependence of polarization-electric field (P-E) hysteresis loops were tested by a Radiant Precision Premier LC ferroelectric material test system attached with a Sigma Model M10 chamber (Sigma Systems Corp., San Diego, CA, USA). Raman spectra upon heating were measured by a Horiba LabRAM HR Evolution Raman spectrometer (HORIBA Instruments Incorporated, Ann Arbor, MI, USA) equipped with a Linkam THMS600 heating/cooling stage [28].

## 3. Results and Discussion

The BCZT-Li ceramics prepared via the citrate method, i.e., calcined between 600 and 700 °C for the 300 °C self-combusted powder and sintered at 1490 and 1500 °C for the ceramics, exhibit phase-pure pseudo-cubic perovskite structure with rather homogenous microstructure morphology and high density [26,27]. All the sintered ceramics present excellent dielectric, ferroelectric and piezoelectric properties, which can be attributed to the MPB effect and the advantages induced by the Li-doping and the wet citrate method [26,27].

The citrate method synthesized BCZT-Li ceramics exhibit enhanced dielectric performance, in which the dielectric response peaks are relatively broad, whereas the frequency dispersion of the dielectric constant is not obvious, and no shift of the *T_C_* temperature occurs between 1 and 300 kHz [26,27]. The dielectric behavior of the BCZT-Li ceramics above *T_C_* can be fitted by both the Curie–Weiss law and the quadratic law [29] but all with slight deviation, presenting their complex dielectric nature correlating with the formation of complex perovskite solid solution [26,27]. The discussion below will use the BCZT-Li ceramics calcined at 675 °C and sintered at 1500 °C prepared via the citrate method due to their excellent comprehensive performance reported by our previous works [26,27].

**Figure 1:**
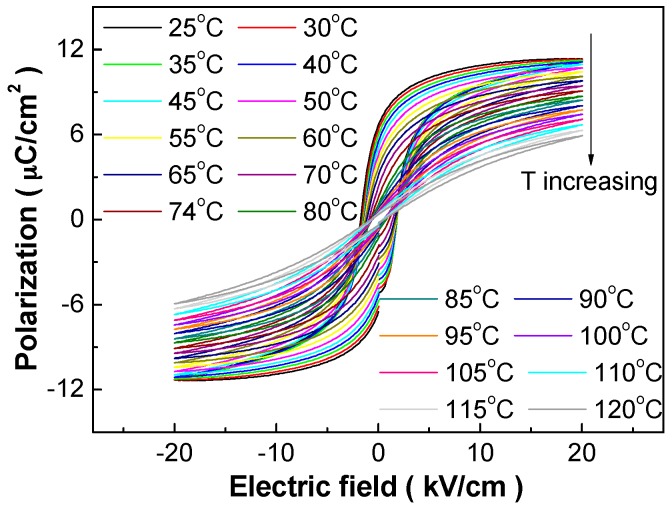
P-E hysteresis loops of the 1 wt % Li-doped (Ba_0.85_Ca_0.15_)(Zr_0.1_Ti_0.9_)O_3_ (BCZT-Li) ceramics measured upon heating between 25 and 120 °C at 1 Hz and maximum electric field of 20 kV/cm.

Figure 1 shows the temperature dependent P-E hysteresis loops of the BCZT-Li ceramics, based on which their electrocaloric effect can be evaluated by the indirect phenomenological method [1,2,3,4]. The citrate method synthesized BCZT-Li ceramics present saturate and symmetric hysteresis loops accompanied by enhanced ferroelectricity as compared with those prepared by the solid-state sintering method [30]. The saturation and squareness of the P-E loops deteriorate greatly, and the loops become slimmer accompanied by the decrease of remnant polarization *P_r_* and coercive field *E_c_* with increasing temperature. Far above the Curie temperature (73 °C), the P-E loops do not show linear behavior, characteristic of pure para-electric phase, and slim loops appear in wide temperature range, implying the existence of ferroelectric clusters or polar nanoregions [31], which corresponds well with the complex perovskite nature (i.e., existence of partial relaxor nature) revealed by the dielectric characterization [26].

**Figure 2:**
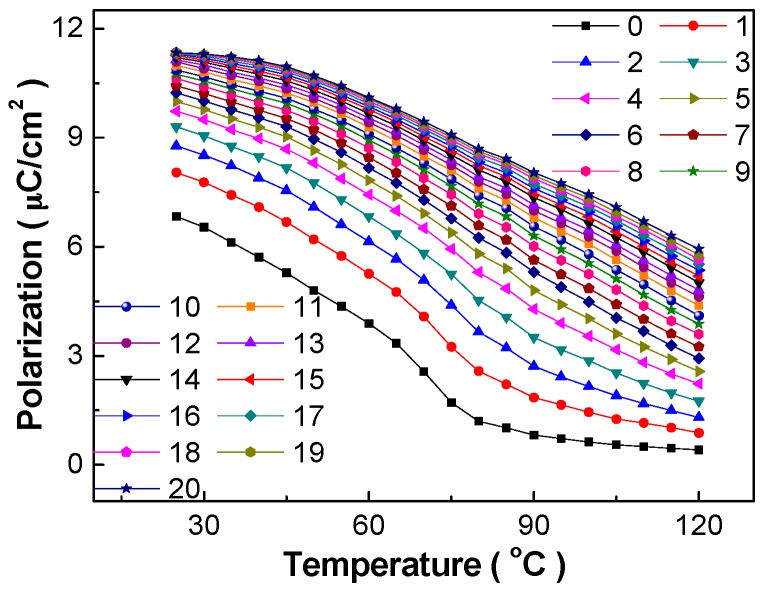
Temperature dependence of polarization of the BCZT-Li ceramics at different electric fields. The unit of the electric field shown by different lines and symbols in the figure is kV/cm.

The change of polarization with temperature under different electric fields is shown in Figure 2 deduced from the first quadrant of the P-E loops of Figure 1. The polarization value decreases apparently with the increase of temperature, in which the *P_r_* value decreases dramatically whereas the spontaneous polarization *P_s_* decreases slowly. Furthermore, the decline magnitude of polarization decreases with the increase of electric field, which will influence the electrocaloric effect.

The electrocaloric effect under electric field was calculated by the thermodynamic relation [1,2,3,4]. Based on the Maxwell equation, the reversible adiabatic electrocaloric temperature change ∆*T* can be deduced indirectly by $\Delta T=-\frac{1}{\rho C}{\int_{E_{1}}^{E_{2}}T\left(\frac{\partial P}{\partial T}\right)}_{E}dE$, where *ρ* is mass density and the *C* is heat capacity [1,2,3,4]. As for the BCZT-Li ceramics, the shape of the P-E hysteresis loops is rather narrow, indicating their small hysteresis loss, therefore, such indirect thermodynamic relation calculation is justified to determine the electrocaloric effect. In an isothermal process, the specific entropy change ∆*S* can be calculated by $\Delta S=-\frac{1}{\rho }{\int_{E_{1}}^{E_{2}}T\left(\frac{\partial P}{\partial T}\right)}_{E}dE$, resulting from the domains rotation under external applied electric field [1,2,3,4]. It can be seen that large polarization gradient (∂P/∂T) will induce excellent electrocaloric effect, i.e., high ∆*T* and ∆*S*.

**Figure 3:**
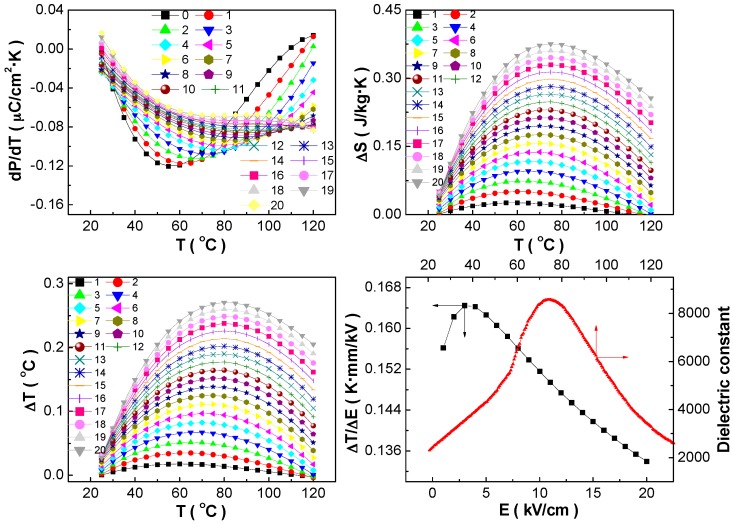
Temperature dependence of *dP*/*dT*, ∆*S* and ∆*T* at different electric fields, and dielectric constant shown the ferroelectric phase transition temperature; and electric field dependent electrocaloric responsivity of the BCZT-Li ceramics. The unit of the electric field shown by different lines and symbols in the figure is kV/cm.

Detailed electrocaloric performance of the BCZT-Li ceramics are shown in Figure 3, in which their ε-T curve without poling is given to show the correlation between the *T_C_* temperature and the maximum electrocaloric effect temperature. The change of *dP*/*dT* depends on temperature and electric field, in which the maximum *dP*/*dT* value appears at zero bias electric filed and 25 °C, being −0.12 μC/cm^2^·K and favorable to obtain large electrocaloric temperature change ∆*T* and adiabatic entropy change ∆*S*.

Considering the temperature-dependent heat capacity, regarding the bulk density as constant, and ignoring the influence of electric field, the calculated ∆*T* and ∆*S* present similar change characteristics, which is useful for searching novel electrocaloric materials. The temperature of the maximum ∆*T* and ∆*S* under different electric fields increases gradually with increasing electric field, around the *T_C_* temperature, which corresponds well with the dielectric performance [26] and confirms that the ferroelectric phase transition can induce large electrocaloric effect. Furthermore, the reduction of the ∆*T* and ∆*S* values induced by the hysteresis loss is small, which can be seen from the area of the P-E hysteresis loops especially at elevated temperatures.

Although the maximum ∆*T* and ∆*S* values can increase further with applying higher external electric filed, the electrocaloric efficiency, representing as *ξ* = ∆*T*_max_/∆*E*_max_ [11], reaches maximum at rather low electric field. Therefore, the electrocaloric figure of merit depends mainly on the external applied electric field, which also induces the change of the ferroelectric phase transition type, i.e., from nearly first-order phase transition to almost second-order one, leading to the decrease of the *ξ* value [4,12]. Due to the small density and heat capacity of the BCZT-Li ceramics, their electrocaloric responsivity, being 0.164 K·mm/kV, can be comparable to the largest values reported of the lead-free piezoelectric ceramics [12,32]. The electrocaloric effect and responsivity values would be even larger if measured by the direct method, in which the discrepancy can be attributed to different polarization switching time needed measured by different methods and disequilibrium unsaturation polarized state for polycrystalline ceramics [32].

**Figure 4:**
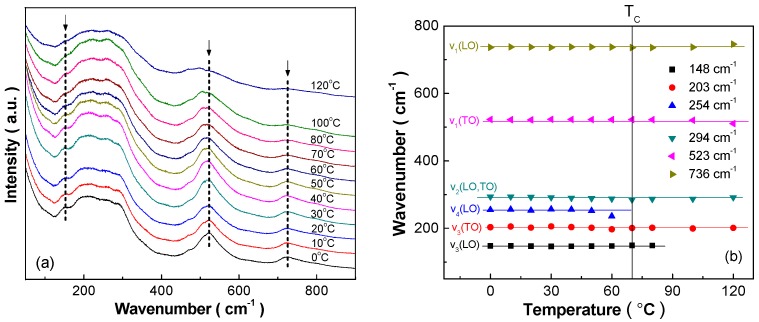
(**a**) Raman spectra of the BCZT-Li ceramics measured upon heating between 0 and 120 °C; (**b**) temperature dependence of wavenumber of different Raman vibration modes of the BCZT-Li ceramics.

**Figure 5:**
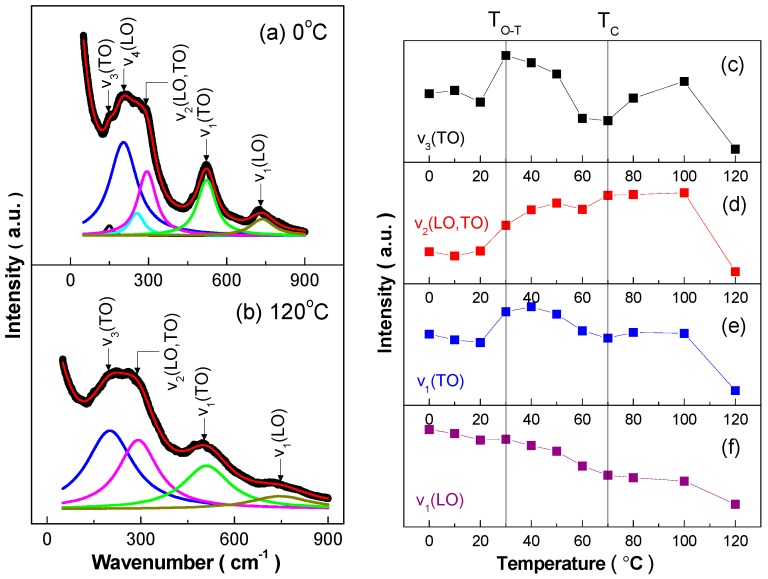
Lorentzian deconvolution peaks of Raman spectra using (**a**) 0 °C and (**b**) 120 °C as examples; (**c**–**f**) temperature-dependent intensity of different Raman modes of the BCZT-Li ceramics upon heating.

The excellent electrocaloric performance of the BCZT-Li ceramics can be attributed to the polymorphic ferroelectric phases coexistence and ferroelectric phase transition, which can be investigated by the Raman spectroscopy due to its sensitivity to crystal lattice vibration, domain configuration dynamics and ferroelectric phase transition [28,33,34] (Figure 4 and Figure 5). As shown in Figure 4a, the Raman modes around 150 cm^−1^ and 740 cm^−1^ wavenumbers weaken gradually, and the Raman mode peak around 520 cm^−1^ wavenumber broadens gradually with the increase of temperature, moreover the intensity of these Raman modes peaks decreases apparently when the temperature approaches to 120 °C.

To analyze the variation of different Raman modes more accurately, Lorentzian deconvolution fitting [35] is necessary as shown in Figure 5a,b using 0 and 120 °C Raman spectra as examples. Based on which the abnormal change of Raman modes peaks location, width, intensity, peak distance and so on can be determined, which provide efficient means to detect the occurrence of ferroelectric phase transition. The v_4_(LO) Raman mode disappears around 70 °C and the v_3_(LO) Raman mode disappears around 80 °C, which attributes to the tetragonal ferroelectric phase to cubic paraelectric phase transition (Figure 4b). At 120 °C, higher than the *T_C_* temperature, four Raman modes, i.e., the v_3_(TO), v_2_(LO,TO), v_1_(TO) and v_1_(LO) modes, still exist, which can be correlated with the existence of ferroelectric clusters or polar nanoregions [31], proving their slight relaxor ferroelectrics characteristic.

The polymorphic ferroelectric phases coexistence can be proven using the abnormal change of the Raman modes intensity as examples (Figure 5c–f). Around 30 °C, the intensity of the v_3_(TO), v_2_(LO,TO) and v_1_(TO) Raman modes increases abruptly, confirming the taking place of another ferroelectric phase transition, i.e., orthorhombic ferroelectric phase to tetragonal ferroelectric phase transition. Therefore, the BCZT-Li ceramics prepared by the citrate method exhibit enhanced electrical properties around room-temperature.

Based on the above discussion, the interplays between polarization, electric field and temperature determine the adiabatic temperature change. To obtain large temperature change, ferroelectric materials with large saturation polarization are desirable, and critical behavior, ferroelectric phase transition or domain transition that occur at operating temperatures will be helpful [36]. To improve the electrocaloric effect further, increasing breakdown electric filed, decreasing polarization fatigue via aliovalent doping, enhancing thermal conductivity via introducing crystallographic texture with low-angle grain boundaries, and lowering *T_C_* toward room-temperature are desired.

## 4. Conclusions

In conclusion, the citrate method synthesized BCZT-Li ceramics present high densification, pure perovskite structure and improved electrical performance, which provides possibility of obtaining large electrocaloric effect. The BCZT-Li ceramics exhibit enhanced ferroelectricity, which exists far above the *T_C_* temperature and can be attributed to the existence of ferroelectric clusters and polar nanoregions. Large ∆*T* and electrocaloric responsivity obtained in the BCZT-Li ceramics can be attributed to the polymorphic ferroelectric phases coexistence and ferroelectric phase transition, which are confirmed by the Raman spectroscopy study.
